# The Glutathione Synthesis Gene *Gclm* Modulates Amphiphilic Polymer-Coated CdSe/ZnS Quantum Dot–Induced Lung Inflammation in Mice

**DOI:** 10.1371/journal.pone.0064165

**Published:** 2013-05-27

**Authors:** Lisa A. McConnachie, Dianne Botta, Collin C. White, Chad S. Weldy, Hui-Wen Wilkerson, Jianbo Yu, Russell Dills, Xiaozhong Yu, William C. Griffith, Elaine M. Faustman, Federico M. Farin, Sean E. Gill, William C. Parks, Xiaoge Hu, Xiaohu Gao, David L. Eaton, Terrance J. Kavanagh

**Affiliations:** 1 Department of Environmental and Occupational Health Sciences, University of Washington, Seattle, Washington, United States of America; 2 Department of Medicine, University of Washington, Seattle, Washington, United States of America; 3 Department of Bioengineering, University of Washington, Seattle, Washington, United States of America; The Ohio State Unversity, United States of America

## Abstract

Quantum dots (QDs) are unique semi-conductor fluorescent nanoparticles with potential uses in a variety of biomedical applications. However, concerns exist regarding their potential toxicity, specifically their capacity to induce oxidative stress and inflammation. In this study we synthesized CdSe/ZnS core/shell QDs with a tri-n-octylphosphine oxide, poly(maleic anhydride-alt-1-tetradecene) (TOPO-PMAT) coating and assessed their effects on lung inflammation in mice. Previously published *in vitro* data demonstrated these TOPO-PMAT QDs cause oxidative stress resulting in increased expression of antioxidant proteins, including heme oxygenase, and the glutathione (GSH) synthesis enzyme glutamate cysteine ligase (GCL). We therefore investigated the effects of these QDs *in vivo* in mice deficient in GSH synthesis (*Gclm* +/− and *Gclm* −/− mice). When mice were exposed via nasal instillation to a TOPO-PMAT QD dose of 6 µg cadmium (Cd) equivalents/kg body weight, neutrophil counts in bronchoalveolar lavage fluid (BALF) increased in both *Gclm* wild-type (+/+) and *Gclm* heterozygous (+/−) mice, whereas *Gclm* null (−/−) mice exhibited no such increase. Levels of the pro-inflammatory cytokines KC and TNFα increased in BALF from *Gclm* +/+ and +/− mice, but not from *Gclm* −/− mice. Analysis of lung Cd levels suggested that QDs were cleared more readily from the lungs of *Gclm* −/− mice. There was no change in matrix metalloproteinase (MMP) activity in any of the mice. However, there was a decrease in whole lung myeloperoxidase (MPO) content in *Gclm* −/− mice, regardless of treatment, relative to untreated *Gclm* +/+ mice. We conclude that in mice TOPO-PMAT QDs have *in vivo* pro-inflammatory properties, and the inflammatory response is dependent on GSH synthesis status. Because there is a common polymorphism in humans that influences GCLM expression, these findings imply that humans with reduced GSH synthesis capabilities may be more susceptible to the pro-inflammatory effects of QDs.

## Introduction

Engineering efforts over the last several years have resulted in stunning advances in the production of nanomaterials that can be utilized for a wide variety of applications. These advances, leading to a broad array of novel nanomaterials, have occurred at such a rapid pace that efforts to understand and characterize the impact of nanomaterial exposure on human health are lagging. One such class, semi-conductor quantum dots (QDs) hold great promise as biological imaging agents, but concerns have been expressed related to their core components which typically consist of potentially toxic heavy metals such as cadmium, selenium, lead and tellurium. Pelley *et al* [1] and Botrill and Green [2] present excellent reviews of recent QD toxicity studies. In these reviews, the authors cite several examples whereby QDs elicit toxicity in a variety of *in vitro* systems, particularly via the generation of reactive oxygen intermediates [3]. Furthermore, previous *in vitro* work in our lab indicated that heme oxygenase-1 is a highly robust and reproducible biomarker of QD exposure indicating activation of oxidative stress signaling across a variety of cell types of either human or murine origin [4].

While *in vitro* studies of QD exposure have indicated the ability to evoke toxicity in a variety of cell culture systems, *in vivo* studies utilizing rodent models may be more informative when attempting to predict potential deleterious human health effects. The potential for QDs to elicit toxicity in the lung is important when considering occupational exposure scenarios, especially during the manufacturing process [5]. Moreover, recent studies indicate that QDs evoke pulmonary inflammation in rodents when delivered via intratracheal instillation or inhalation [6], [7]. QDs are of concern when considering pulmonary exposure not only because of their ability to incite inflammation but also of their aforementioned heavy metal core constituents. While chemical modifications to the surface are known to play a large role in QD toxicity, it is also important to consider possible effects associated with metal release upon QD degradation. Cadmium, a potent oxidant, exhibits a long half-life in human kidney and liver tissue, and is a well-characterized renal toxicant [8]. The toxicity of Cd is modulated, in part, by glutathione (GSH) due to the propensity of free Cd to deplete thiols such as GSH through reactive oxygen species generation and/or direct binding and sequestration [9]. Furthermore, GSH is known to modulate inflammatory responses and is generally protective against inflammatory pathologies [10], [11]. To this end, recent work in our laboratory has indicated that GSH status plays a pivotal role in determining the degree of inflammatory response in the lung following exposure to diesel exhaust particulate [12]. In that report, transgenic mice with partially compromised capacity for GSH synthesis exhibited a significantly higher degree of neutrophil influx into the lung following diesel particulate exposure relative to mice with normal GSH synthesis capacity.

Although the amphiphilic polymer coated tri-n-octylphosphine oxide, poly(maleic anhydride-alt-1-tetradecene) (TOPO-PMAT) QDs employed in the present study are designed to be stable [13], [14], there is nevertheless the possibility they will degrade and release free Cd^+2^ causing downstream toxicity via the generation of reactive oxygen species and GSH depletion. Therefore, the goal of the present study was to examine the role of GSH in modulating TOPO-PMAT QD pro-inflammatory properties in mice when dosed via nasal instillation. This particular route of administration is a useful screening paradigm to identify materials that have the potential to cause toxicity and inflammation in the lung following inhalation exposure.

The first and rate-limiting step in GSH biosynthesis is the ligation of glutamate and cysteine to form γ-glutamylcysteine (γ-GC). This reaction is catalyzed by glutamate-cysteine ligase (GCL), a heterodimer composed of catalytic and modifier subunits. Mice lacking the modifier subunit of GCL (Gclm −/−), have a dramatically compromised ability to synthesize GSH in all tissues relative to their wild-type counterparts (Gclm +/+). Mice heterozygous for Gclm (+/−) have slightly lower levels of GSH than wild type Gclm +/+ mice, but can be sensitive to certain toxic exposures, presumabely because they are less able to rapidly re-synthesize GSH following challenge [12], [15].

To investigate the role of GSH in modulating QD induced inflammation and toxicity, we exposed mice of all three *Gclm* genotypes (+/+, +/−, and −/−) to TOPO-PMAT QDs and examined markers of pulmonary inflammation and toxicity at various time-points up to 24-hours following exposure. Overall, we found that the degree of inflammation, as measured by neutrophil influx and pro-inflammatory cytokines, was highly dependent on *Gclm* genotype, and hence GSH levels. Collectively, these results indicate that GSH status plays a potential role in determining the toxicity and disposition of TOPO-PMAT QDs. Because there are relatively common functional genetic polymorphisms in the human *GCLM* gene that can limit the expression of GCLM and thus re-synthesis of GSH under conditions of oxidative challenge, these findings also suggest caution is warranted when considering potential human exposures to such nanomaterials.

## Materials and Methods

### Quantum Dot Synthesis and Characterization

**Figure 1:**
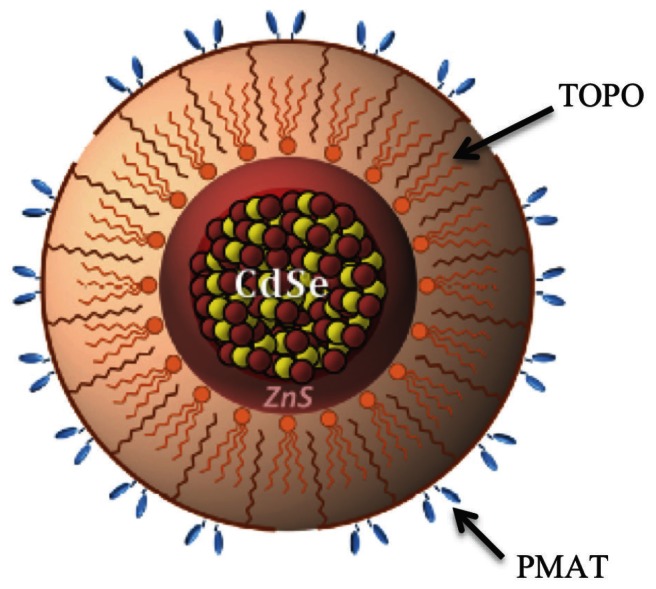
Schematic representation of a TOPO-PMAT quantum dot. The central sphere represents the CdSe/ZnS core/cap structure, surrounded next by tri-n-octylphosphine oxide (TOPO), and then the poly(maleic anhydride-alt-1-tetradecene (PMAT) polymer coating. (Modified from [42]).

We utilized TOPO-PMAT coated CdSe/ZnS core/shell QDs for all studies. The characteristics of these QDs have been described previously [4]. In brief, CdSe/ZnS core/shell quantum dots coated with tri-n-octylphosphine oxide (TOPO) were supplied by Ocean Nanosciences (Springdale, AR). They were further coated with poly(maleic anhydride-alt-tetradecene) (PMAT) (Sigma Aldrich, St. Louis MO). Figure 1 describes the structure and composition of the TOPO-PMAT QDs. Immediately prior to dosing, the QDs were vortexed and diluted in sterile injection grade 0.9% sodium chloride to a final concentration of 5 nM. The QDs were tested for endotoxin using a Kinetic-QCL™ LAL Assay (Lonza, Inc., Walkersville, MD).

### Mice

All animal experiments were conducted in accordance with the National Institutes of Health Guide for the Use and Care of Laboratory Animals and were approved by the University of Washington Institutional Animal Care and Use Committee (protocol #2384-08). All efforts were taken to minimize suffering. Male *Gclm* +/+, +/−, and −/− mice, previously backcrossed onto a C57BL/6 background [15], were used for all studies. Mice were housed in a modified specific pathogen free vivarium on a 12-hour light/dark cycle and allowed access to chow and water *ad libitum*.

### Quantum Dot Treatments

Eight to 24 week old mice (median age 9 weeks) of all three *Gclm* genotypes were randomly assigned to receive either saline or TOPO-PMAT QD (1.53 µL/gm body weight of a 5 nM solution; 6 µg Cd equivalents/kg BW) via nasal instillation. Nasal instillation was performed following induction of light anesthesia (intraperitoneal injection of 0.01 mL/g body weight of a 0.44 mg/mL Xylazine, 6.5 mg/mL Ketamine solution in sterile saline) and by slowly depositing half the dose of TOPO-PMAT QD solution (or saline vehicle) into each nostril. Mice were held in an upright position following dosing (for one minute) to ensure complete inspiration of saline or TOPO-PMAT QDs. At specified time-points (30 min, 1, 3, 8 or 24 hours following nasal instillation) mice were sacrificed by CO_2_ narcosis followed by cervical dislocation. After collecting bronchoalveolar lavage fluid (described below) the lungs were inflated with 1 mL of Tissue-Tek* CRYO-OCT cutting compound (Fischer Scientific, Pittsburgh, PA), and the left lobe was resected and snap frozen in liquid nitrogen. The right lung lobes were embedded in OCT for cyrosectioning. Portions of the left lobe were then processed for Cd analysis at all of the above listed time points, and the remaining assays described below were performed on samples collected at the 8-hour time point.

### Bronchoalveolar Lavage

Bronchoalveolar lavage (BAL) was performed as previously described [12]. In brief, after lavage, collected cells were pelleted by centrifugation and prepared for flow cytometry analysis to quantitate the percentage of neutrophils present. The supernatant was reserved for cytokine analyses.

### Flow Cytometry

We utilized a Beckman-Coulter Altra fluorescence activated cell sorter (Beckman-Coulter, Miami, FL) to quantitatively assess the percentage of neutrophils in BAL by flow cytometry. Cells obtained from BAL were stained with anti-F4/80 conjugated to Alexafluor 488 (eBioscience, San Diego, CA), and biotinylated anti-mouse Ly6G/Ly6C (Gr1) (BioLegend, San Diego, CA) followed by streptavidin Alexafluor 350 (Invitrogen, Carlsbad, CA). Neutrophils were identified as cells having low F4/80 and high Gr1 staining as indicated on green versus blue fluorescence intensity bivariate dot-plots.

### Total Lung Glutathione Determination

Total lung glutathione (GSH) was determined as previously described [12]. In brief, clarified lung homogenates from mice (8 hours post-exposure) were prepared from snap frozen lung tissue. GSH was derivatized with napthelene-2,3-dicarboxaldehyde and the relative fluorescence intensity of derivatized products was determined. GSH levels were determined by interpolation on a GSH standard curve prepared identically to test samples. All determinations were carried out in triplicate.

### Fluorogenic 5′ Nuclease-based Assay and Quantitative RT-PCR

The Functional Genomics Laboratory at the University of Washington developed fluorogenic 5′ nuclease-based assays to quantitate the mRNA levels of specific genes. Briefly, reverse transcription was performed according to the manufacturer’s established protocol using 1 ug of total RNA and the SuperScript® III First-Strand Synthesis System (Invitrogen, Carlsbad, CA). For gene expression measurements, 2****µL of cDNA were included in a PCR reaction (12 µL final volume) that also consisted of the appropriate forward (FP) and reverse (RP) primers, probes and TaqMan Gene Expression Master Mix (Applied Biosystems Inc., Foster City, CA). The PCR primers and the dual-labeled probes for the genes were designed using ABI Primer Express v.1.5 software (Applied Biosystems). Several genes were analyzed using the ABI inventoried TaqMan® Gene Expression Assays mix according to the manufacturer’s protocol (Applied Biosystems). All oligonucleotide sequences are listed in Table S1.

Amplification and detection of PCR amplicons were performed with the ABI PRISM 7900 system (Applied Biosystems) with the following PCR reaction profile: 1 cycle of 95°C for 10 min., followed by 40 cycles of 95°C for 30 sec, and 62°C for 1 min. GAPDH amplification plots derived from serial dilutions of an established reference sample were used to create a linear regression formula in order to calculate expression levels, and GAPDH gene expression levels were utilized as an internal control to normalize the data.

### Cadmium Content

To determine Cd content, lung tissue was initially subjected to microwave digestion in concentrated nitric acid. Because the lungs were instilled with OCT fixative, we were unable to obtain an accurate mass of the tissue for normalization purposes. Therefore, it was necessary to normalize cadmium content to another metal that was invariant with respect to genotype or treatment. Following a survey of metals present in multiple lung samples from mice of all *Gclm* genotypes and treatments, we determined that molybdenum was invariant (data not shown). Thus we used tissue molybdenum levels for normalization. Cadmium and molybdenum content analyses were performed using an inductively coupled plasma mass spectrometer (ICP-MS) following EPA Method 6020A with an Agilent 7500ce ICP-MS.

### Myeloperoxidase Content

Myeloperoxidase (MPO) content in lung homogenates was assessed by ELISA for the 8-hour time point and performed as previously described [12]. Briefly, lungs were homogenized in tissue lysis buffer (Sigma- Aldrich) with proteinase inhibitor (Roche Applied Science, Indianapolis, IN, USA), aliquoted, and snap frozen in liquid nitrogen. A 96-well microtiter plate was loaded with capture antibody overnight at room temperature and after blocking with 5% BSA in PBS for 1 hour, incubated with sample or standard (0.4 to 250 ng/mL) overnight at 4°C. After washing, wells were incubated with biotinylated detection antibody. After 2 hours, wells were washed and then incubated with Streptavidin conjugated to horseradish peroxidase for 20 minutes. Wells were washed again, and TMB Substrate (Sigma-Aldrich) was added to each well and incubated for 5–20 minutes. One hundred µL of 0.5 M H_2_SO_4_ were used to stop the reaction, and plates were read at 450 nm using a spectrophotometric plate reader (Synergy 4 Multi-Mode, BioTek, Winooski, VT).

### Matrix Metalloproteinase Activity

Matrix metalloproteinase (MMP) activity in lung homogenates from the 8-hour time point was measured as previously described [16]. Total MMP activity was determined with the OmniMMP fluorogenic substrate (P-126, Biomol International, Plymouth Meeting PA).

### Multiplex Bead-based Cytokine Assays for CXCL1 (KC), TNFα, MIP2 and MCP-1

Mouse lavage fluid samples were analyzed in the Center for Ecogenetics and Environmental Health Functional Genomics Laboratory at the University of Washington using the MILLIPLEX MAP Mouse bead-based multiplex cytokine assays (Millipore, Billerica, MA) according to the manufacturer’s instructions. Briefly, 25 µL of each standard from a series of 4-fold dilutions and samples were incubated with the target capturing beads on a 96-well plate for 16 hours at 4°C, followed by one hour of incubation with specific biotinylated detection antibodies at room temperature. Next, Streptavidin-Phycoerythrin was added to each well and incubated for 30 minutes at room temperature. Filtration and washing were incorporated after each incubation step with wash buffer using a vacuum manifold. Samples were resuspended in sheath fluid prior to reading them on a Luminex 100™ (Austin, TX) suspension array reader. Each sample was analyzed in duplicate.

### Toxicokinetic Modeling

Cd contents were normalized to tissue molybdenum content (Mo). The average concentration of Cd in the lung tissue from PMAT-TOPO QD treated mice (N = 5–7) at each time-point for the toxicokinetic parameter calculations at 0, 0.5,1, 3, 8, and 24 h. The calculation of the compartmental toxicokinetic model was conducted with PKSolver [17]. One compartment toxicokinetic models were found to be the best-fit for simulating lung Cd content.

### Statistical Analyses

Data were analyzed using two-way analysis of variance (ANOVA) for genotypes, treatments, and interactions. Quantal- quantal plots were used to check that residuals from the 2-way ANOVA were approximately normally distributed. Post-hoc comparisons were corrected for multiple testing by using a multivariate t-distribution to compute corrected p-values based upon the variance-covariance matrix for the set of contrasts describing the post-hoc comparisons [18]. The post hoc tests were to detect significant increases in neutrophils, gene expression, protein, activity, or content after treatment with QDs for each Gclm genotype.

## Results

### TOPO-PMAT Quantum Dot Characterization

When measured by dynamic light scattering, the TOPO-PMAT QDs used in this study had a hydrodynamic diameter of 12.7±0.5 nm and a core diameter of 6.8±0.5 nm. Endotoxin contamination of these preparations was found to be negligible (0.013 EU/ml for the instilled QD solution). More in-depth information regarding the physical and chemical characteristics of these particular QDs is presented in McConnachie *et al* [4].

### Neutrophil Influx into the Lungs as Assessed by Bronchoalveolar Lavage

**Figure 2:**
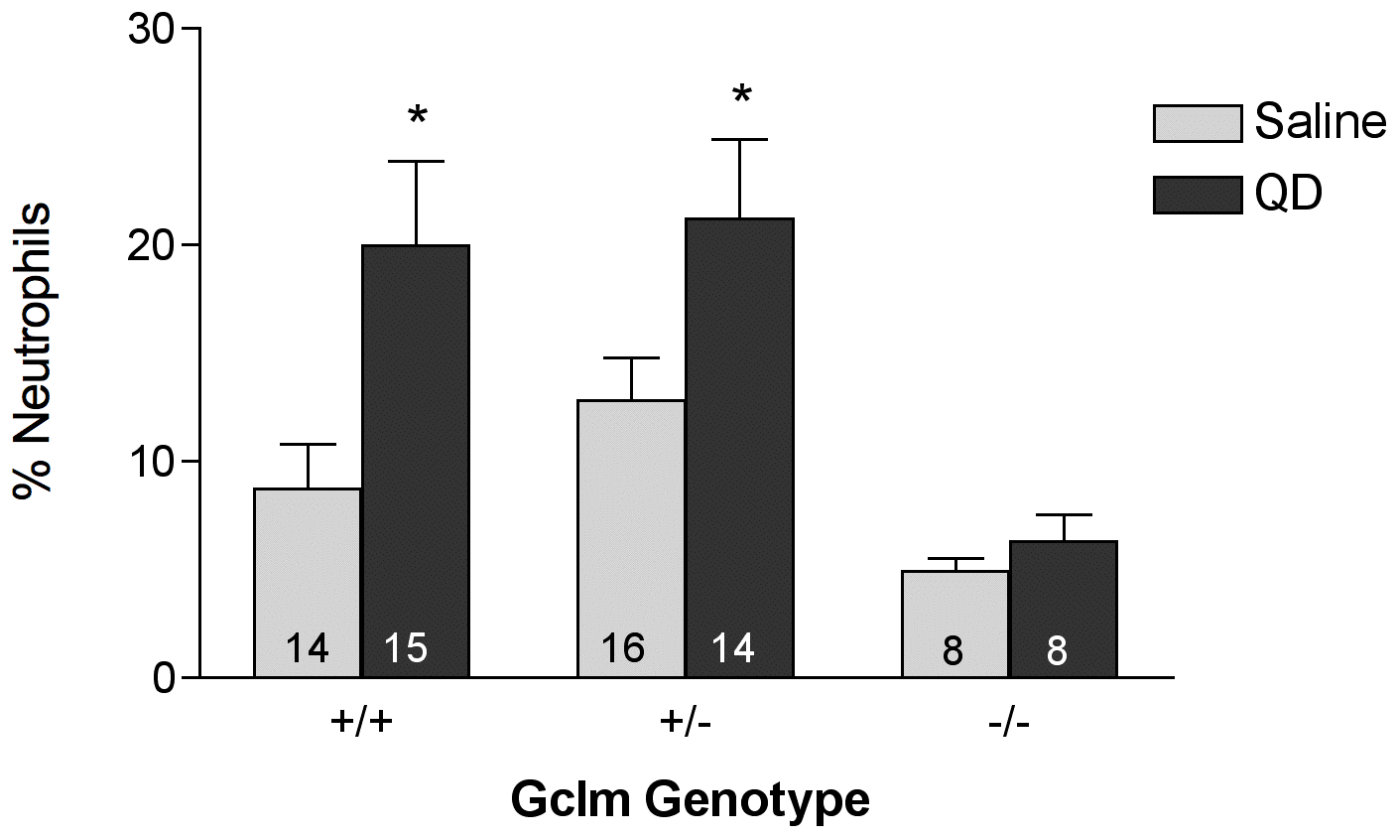
% Neutrophil influx into the bronchoalveolar lavage fluid. Data represent the mean ± S.E.M. for % neutrophils (cells exhibiting low F4/80 and high Gr1 staining) present in the bronchoalveolar lavage fluid as measured by flow cytometry 8 hours following saline or TOPO-PMAT QD administration. * = p<0.05 relative to saline treated mice. The number of replicates is indicated in each bar.

To investigate the potential pro-inflammatory properties of TOPO-PMAT QDs when dosed via nasal instillation, we determined the percentage of neutrophils present in bronchoalveolar lavage fluid (BALF) 8 hours after QD administration. Mice instilled with an equal volume of saline served as vehicle controls. QD instillation elicited a strong and statistically significant neutrophil influx in both *Gclm* +/+ and Gclm +/− mice at 8 hours (p<0.05 relative to saline treated controls of the same genotype). Importantly, TOPO-PMAT QD treatment failed to elicit any such neutrophil response in the *Gclm* −/− mice (Figure 2). The resistance to neutrophil influx in *Gclm* −/− mice may indicate that they are unable to mount an inflammatory response to QD exposure or that there are differences in QD disposition in the lungs of these mice.

### Lung Glutathione Content

**Figure 3:**
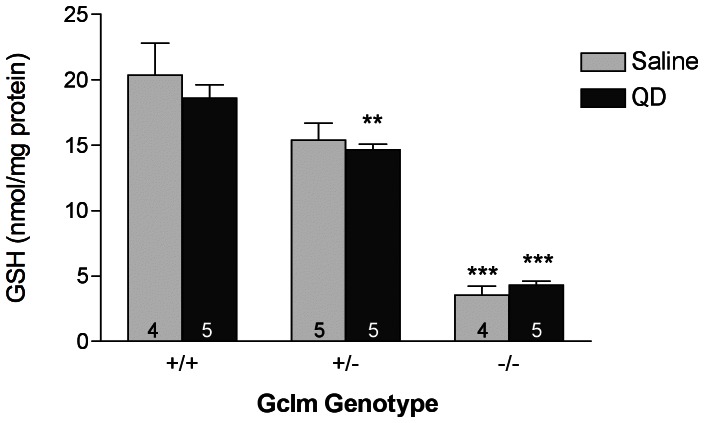
Lung glutathione content. Total glutathione was measured in clarified lung homogenates 8 hours following saline or TOPO-PMAT QD administration. Data represent the mean ± S.E.M., ** = p<0.01 and *** = p<0.001 relative to treatment-matched *Gclm* +/+ mice as determined by Students t-test. The number of replicates is indicated in each bar.

Cadmium is a core constituent of the TOPO-PMAT QDs and may be released upon their degradation. Furthermore, cadmium toxicity is modulated in part by GSH levels [19], [20]. Additionally, oxidative stress and subsequent GSH depletion may be an integral component of the pro-inflammatory potential of heavy metal containing nanoparticles [3], [21]. For these reasons, we were interested in the effect of TOPO-PMAT QD exposure on lung GSH levels. We determined the lung GSH content 8 hours following nasal instillation of either saline or TOPO-PMAT QDs (Figure 3). TOPO-PMAT QD treatment had no effect on lung GSH levels, relative to saline treatment, regardless of genotype. As expected, the levels of GSH present in the lungs of saline-treated *Gclm* −/− and *Gclm* +/− mice were approximately 17% and 77%, respectively, of that in saline-treated *Gclm* +/+ mice. These data are in agreement with previously reported values for these mice [12], [15].

### Real Time PCR Assessment of Stress Response and Inflammatory Cytokine mRNA Expression

**Figure 4:**
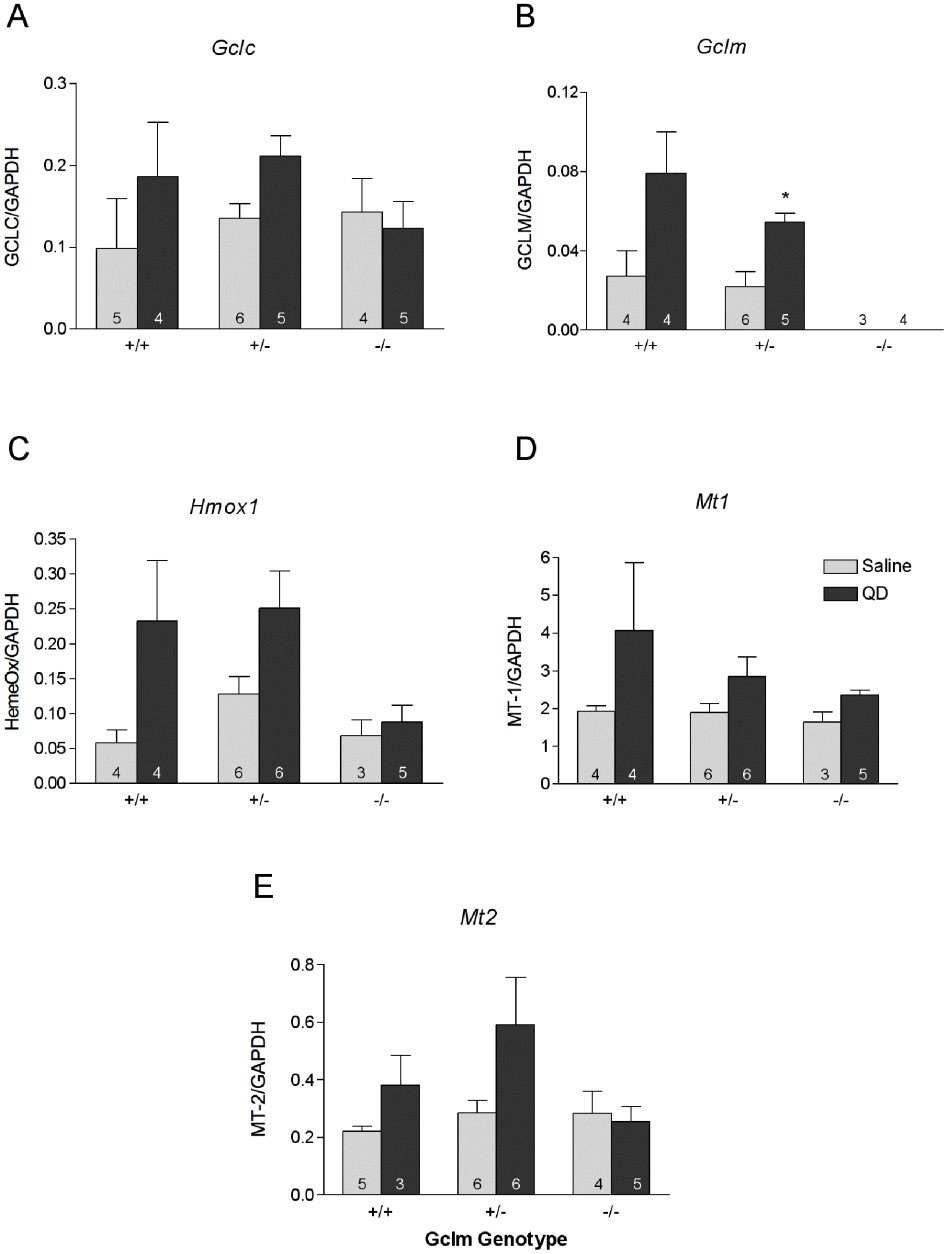
mRNA expression of stress-response genes as measured by qRT-PCR. The effects of saline and TOPO-PMAT QD administration on A) *Gclc*, B) *Gclm*, C) *Hmox1*, D) *Mt1* and E) *Mt2* mRNA expression are shown. Data represent the mean ± S.E.M., * = p<0.05 relative to saline treated *Gclm* +/− mice. The number of replicates is indicated in each bar.

We analyzed the mRNA expression level of genes involved in both oxidative stress signaling and inflammatory pathways (Figure 4). Genes in the former group were glutamate cysteine ligase catalytic subunit (*Gclc)*, glutamate cysteine ligase modifier subunit (*Gclm)*, heme oxygenase-1 (*Hmox1*) and metallothionein-1/2 (*Mt1/2*). While there was a trend towards increasing *Gclc* expression with QD exposure in +/+ and +/− mice, this did not achieve statistical significance. There was no difference in *Gclc* expression after QD treatment in −/− mice relative to saline-treated controls. Conversely, QD-treatment resulted in marked increases in *Gclm* expression in +/− mice (p<0.05) and a suggestion of an increase in Gclm +/+ mice, relative to the saline-treated control groups. As expected, *Gclm* −/− mice had no detectable levels of *Gclm* mRNA in either the saline- or QD-treated groups. This pattern of induction in *Gclm* +/+ and +/− mice with QD treatment was also observed for *Hmox1* mRNA (although not statistically significant). *Gclm* −/− mice, on the other hand, did not exhibit any perturbation in *Hmox1* expression following QD treatment. In addition, there were treatment related changes noted in *Mt1* expression by 2-way ANOVA analysis. It should also be noted that there appeared to be an inverse trend for *Mt1* expression relative to *Gclm* copy number. *Mt2* expression changes were minimal and did not achieve statistical significance, although there was a trend towards increasing expression with TOPO-PMAT QD treatment. Taken together, the data from the stress-response gene expression analyses indicate mild to moderate up-regulation of these pathways associated with TOPO-PMAT QD exposure in the *Gclm* +/+ and +/− mice but no effects in the *Gclm* −/− mice.

**Figure 5:**
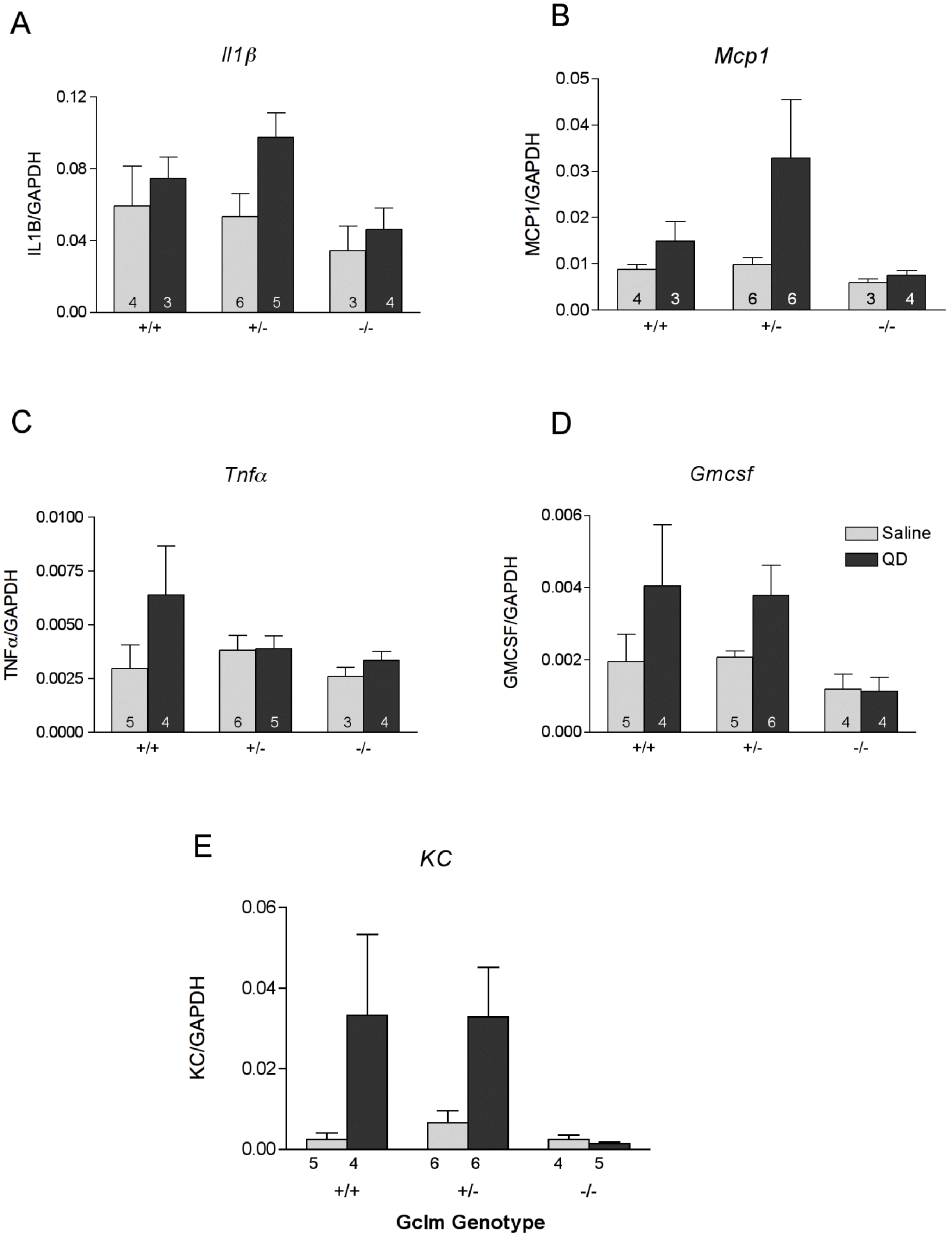
mRNA expression of inflammatory cytokine genes as measured by qRT-PCR. The effects of saline or TOPO-PMAT QD administration on A) *Il1β*, B) *Mcp1*, C) *Tnfα*, D) *Gmcsf* and E) *KC* mRNA expression are shown. Data represent the mean ± S.E.M. of the indicated number of replicates in each bar.

We analyzed mRNA expression for the following genes involved in inflammation: interleukin-1β (*Il1β)*, monocyte chemotactic protein-1 (*Mcp1)*, tumor necrosis factor-α (*Tnf*α), granulocyte-macrophage colony stimulating factor (*Gmcsf*), and keratinocyte derived cytokine (*KC, Cxcl1*) (Figure 5). Although there was a suggestion of an increase in several of these mRNAs relative to saline-treated controls in *Gclm* +/+ and *Gclm* +/− mice, especially with respect to KC levels, these did not achieve statistical significance. Furthermore, as observed with the % neutrophil data and stress response gene analyses, *Gclm*−/− mice were non-responsive to QD exposure.

### Assessment of KC and TNFα Protein Concentrations in Bronchoalveolar Lavage Fluid

**Figure 6:**
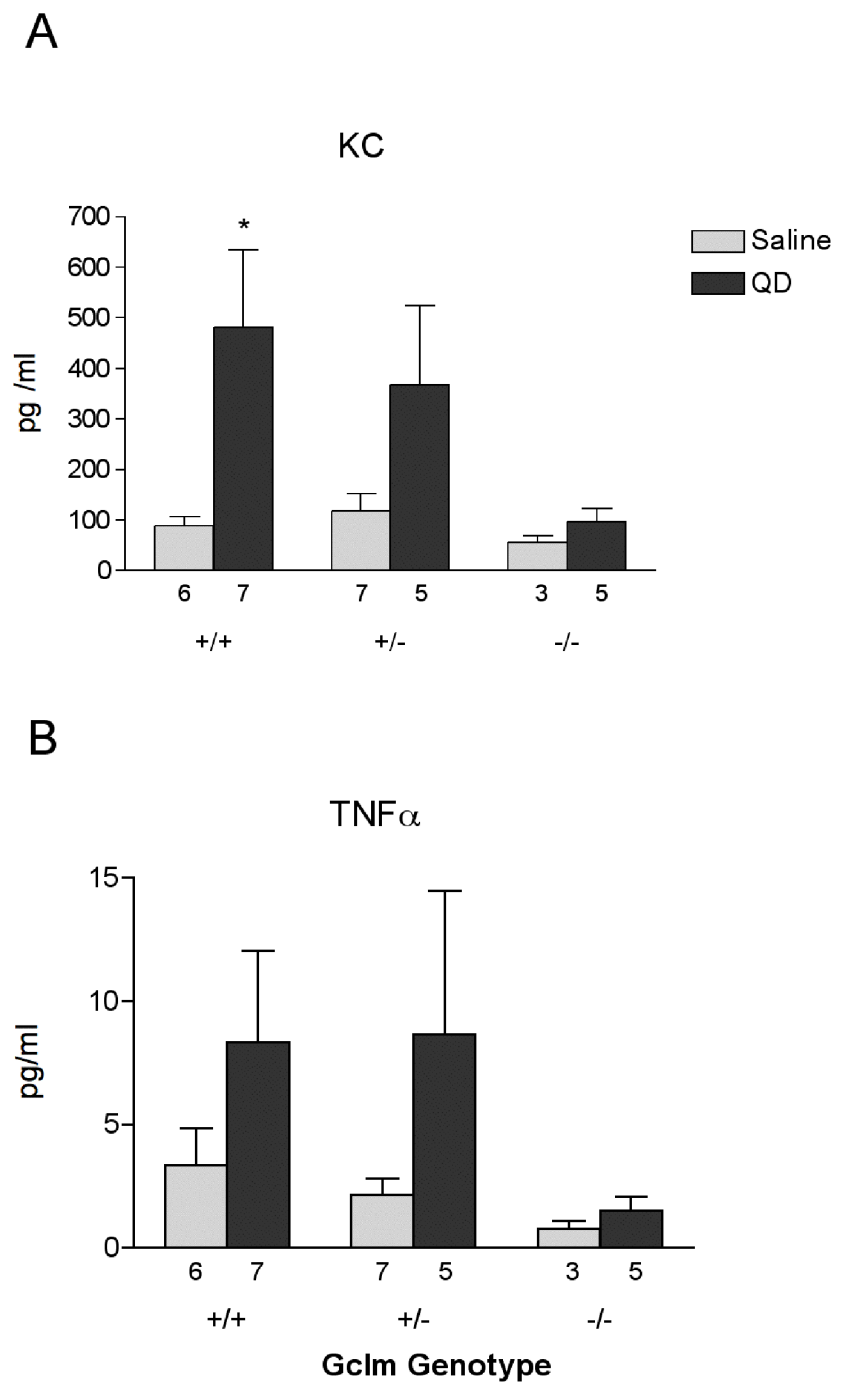
Inflammatory cytokine protein expression in lung tissue. The effect of saline or TOPO-PMAT QD administration on A) KC and B) TNFα protein levels as assessed in a cytokine array panel. Data represent the mean ± S.E.M. of the indicated number of replicates in each bar. * = p<0.05 relative to saline treated mice.

In order to further clarify the potential pro-inflammatory properties of the TOPO-PMAT QD, we also analyzed the level of inflammatory cytokines keratinocyte-derived cytokine (KC) and TNFα protein present in the BAL fluid 8 hours after either saline or TOPO-PMAT QD instillation (Figure 6). QD treatment resulted in an increase in KC levels in *Gclm* +/+ mice relative to treatment with saline alone (p<0.05). A similar trend was observed for TNFα levels, although this did not achieve statistical significance. In contrast, *Gclm* −/− mice exhibited a more muted KC and TNFα response to QD treatment. There were no significant differences in these cytokines across *Gclm* genotypes for the saline-treated mice, indicating a similar low-level response to saline regardless of GSH status. In general, the findings of this analysis are consistent with the neutrophil data presented in Figure 2.

### Matrix Metalloproteinase Activity and Myeloperoxidase Content

**Figure 7:**
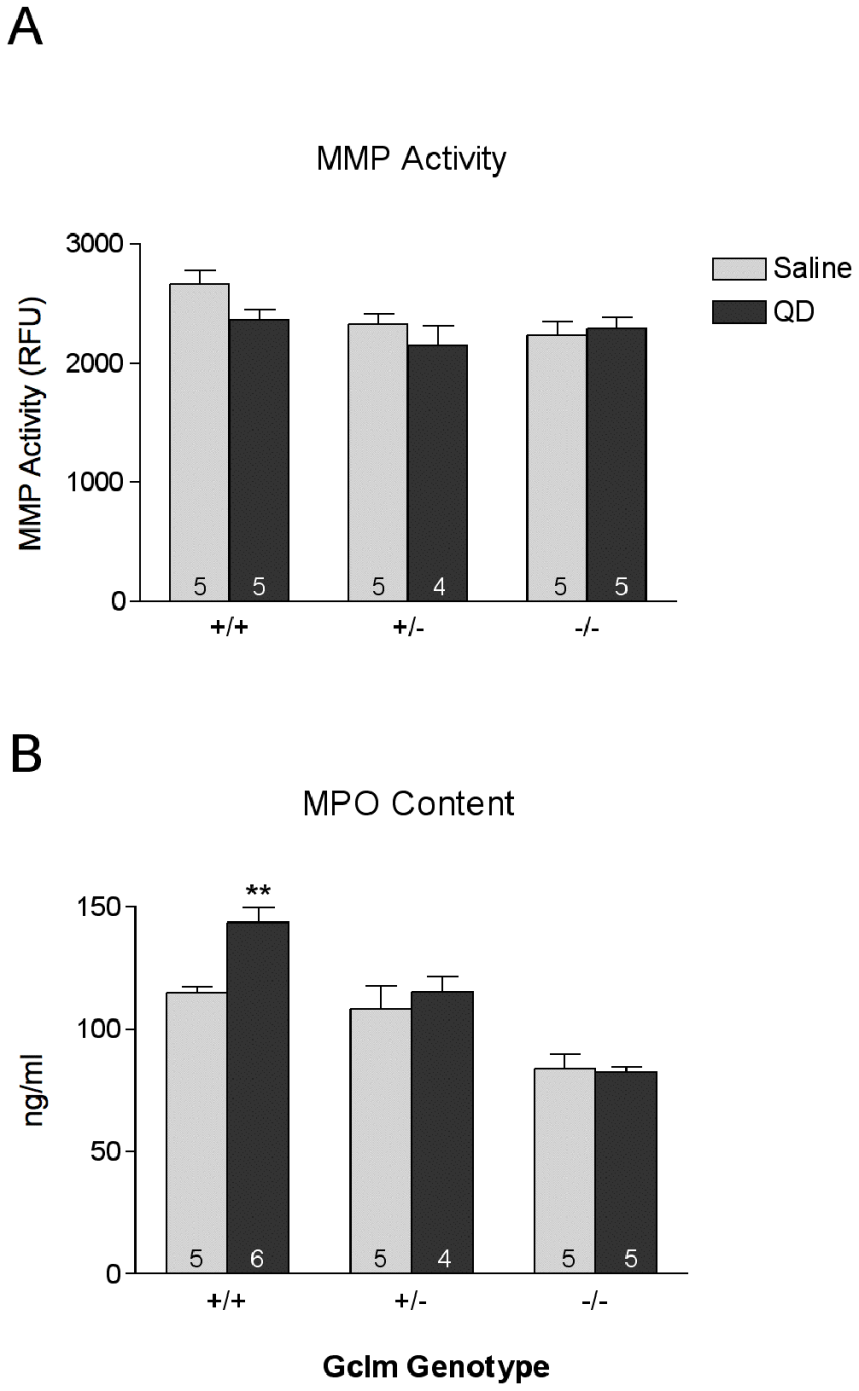
Lung matrix metalloproteinase activity and myeloperoxidase content. The effect of TOPO-PMAT QD administration on A) **matrix metalloproteinase (**MMP) activity and B) **myeloperoxidase** (MPO) content. Data represent the mean ± S.E.M. of 4 replicates ** = p<0.01 relative to saline treated mice.

Matrix metalloproteinase (MMP) activity serves to promote neutrophil influx via shedding of extracellular matrix proteins, facilitating neutrophil chemotaxis into the lung. Thus, an increase in MMP activity may facilitate neutrophil recruitment into the lung following QD treatment. Additionally, neutrophils express high levels of myeloperoxidase (MPO), which can be considered a biomarker of neutrophil presence in the lung. Analysis of lung MMP activity and MPO levels revealed few significant effects of TOPO-PMAT QD instillation in lung tissue (Figure 7). A notable exception is observed for *Gclm* +/+ mice where we observed that QD exposure led to a significant increase in MPO content. This finding is consistent with the % neutrophil data presented in Figure 2. Interestingly, the level of MPO was lower in *Gclm* −/− mice, relative to *Gclm* +/+ mice, regardless of treatment, which is consistent with the lower levels of neutrophils in the BAL fluid from these mice.

### Toxicokinetic Modeling of Cadmium Disposition in Lung Tissue

**Figure 8:**
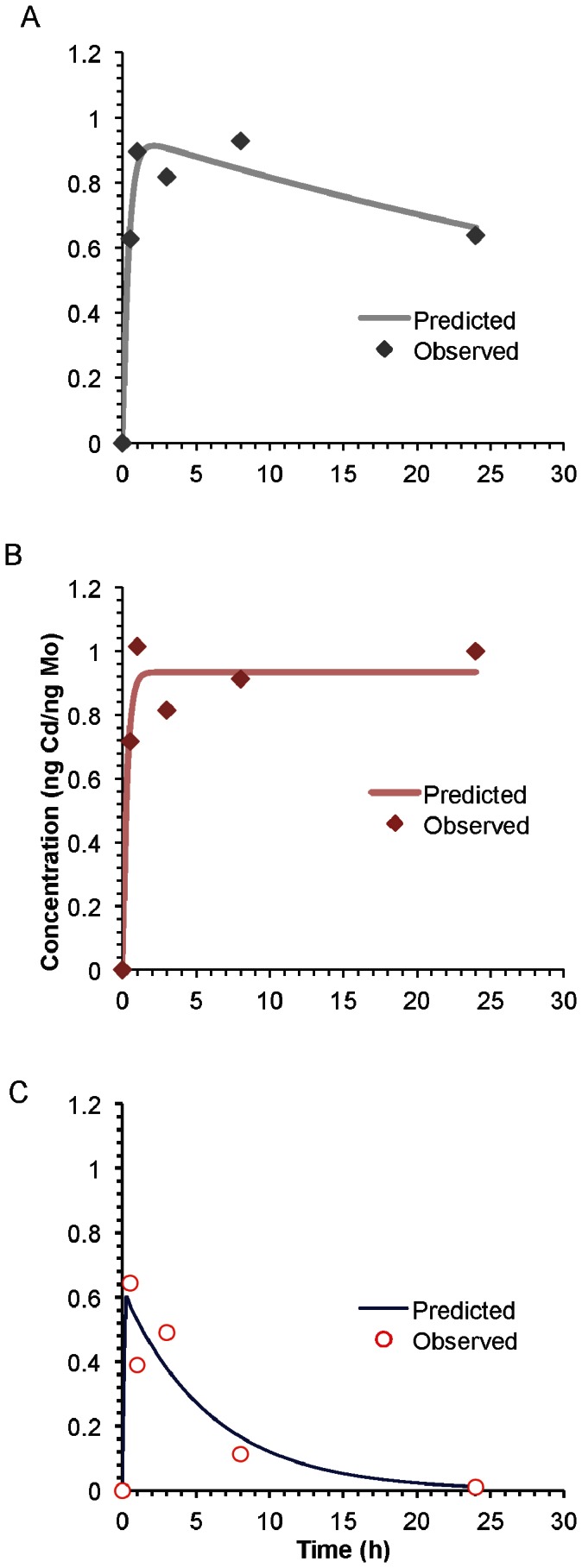
Toxicokinetic modeling of TOPO-PMAT QD disposition. Lung cadmium content was determined by inductively coupled plasma-mass spectrometry (ICP-MS) analysis, and TOPO-PMAT QD disposition was modeled using PKSolver as described in Methods. A one-compartment model resulted in the best fit of simulated QD disposition curves relative to measured Cd content.

The parameters in Table 1 are for a one compartment model for the lung concentration of Cd. The parameters *A* and *alpha* are the uptake concentration and uptake rate for Cd entering the lung, and the parameter *beta* is the rate at which it leaves the lung:
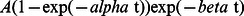
where t is the time post exposure in hours. The results of applying these rates in a one compartment lung model are shown in Figure 8 and provided an excellent fit for lung Cd data; the r^2^ values between the observed and predicted Cd content are 0.99, 0.99 and 0.95, for *Gclm* +/+, +/− and −/− mice, respectively. The kinetic rate constants derived from the modeling reveal again several striking differences between genotypes suggesting *Gclm* status plays an integral role in the disposition of TOPO-PMAT QDs. There was rapid deposition of Cd for all three genotypes and we could not distinguish between the delivery rates, *alpha,* which had an deposition half time of 12.6 minutes. The deposited concentration of Cd, *A,* appeared to be lower for the Gclm −/− mice with a value of 0.62 ng Cd/mg Mo compared to 0.95 and 0.93 ng Cd/mg Mo for the Gclm +/+ and +/− mice. The rate of clearance of Cd from the lung, *beta,* was much more rapid for the Gclm −/− mice with a half life of 4.6 hours compared to the Gclm +/+ and +/− genotypes with half lives of 46 hours and over 400 hours respectively.

**Table 1:** Toxicokinetic parameters used to model TOPO-PMAT QD disposition.

		Gclm Genotype		
Parameter	Units	+/+	+/−	−/−
*A*	ng Cd/ng Mo	0.95	0.93	0.62
*alpha*	1/h	3.3	3.3	3.3
*beta*	1/h	1.5E-02	1.5E-03	1. 5E-01

## Discussion

To investigate the role of GSH in TOPO-PMAT QD induced pulmonary toxicity, we utilized mice with partially or severely diminished GSH synthesis capacities (*Gclm* +/− and *Gclm* −/−, respectively). In these studies we employed nasal instillation to model inhalation exposure, an important route of exposure when considering exposures in occupational settings. Several interesting findings arose from these studies, namely that (1) *Gclm* status is a major determinant of the degree of pulmonary inflammation following TOPO-PMAT QD exposure via nasal instillation and (2) despite all mice receiving equivalent doses of TOPO-PMAT QDs, there are large differences in lung Cd disposition kinetics which were also dependent on GSH status.

The degree of neutrophil influx into the airways of *Gclm* −/− mice was much lower than mice with relatively normal GSH levels 8 hours following TOPO-PMAT QD exposure. This finding may seem counter-intuitive, in that *Gclm* −/− mice would be expected to be more, rather than less sensitive to the potential oxidative properties of the TOPO-PMAT QDs. It has been shown that GSH status is an important determinant of both innate and acquired immunity in the lung [10], [22], [23]. Interestingly, Villa and colleagues [24], have shown that pharmacological depletion of GSH in mice was associated with a decreased influx of neutrophils into the peritoneal space in a sepsis model (cecal ligation and puncture), while simultaneously increasing the numbers of neutrophils present in the lung. Alternatively, administering GSH-ethyl ester to counteract LPS-induced GSH depletion resulted in protection from LPS-induced lung injury [25]. These effects are likely attributable to differences in local conditions in these organs that govern neutrophil trafficking and may be related to the expression of molecules that are critical for extravasation of neutrophils (e.g. intercellular adhesion molecule-1 and its cognate receptor on neutrophils, CD18) and/or gradients of chemokines/cytokines known to attract neutrophils to the sites of inflammation (e.g. KC, TNFα, IL1β, GMCSF, MIP2 and MCP1). In turn, the expression of these factors is to a large extent governed by the activation of transcription factors such as NFκB and AP-1. Interestingly, while moderate oxidation can increase NFκB signaling [26], which may be dependent on S-glutathionylation of IKKβ [27] or IκB [28], further oxidation can diminish the binding of NFκB to DNA [29]. This inactivation of NFκB under strongly oxidizing conditions has been demonstrated whereby severely depleted GSH can suppress NFκB signaling because of oxidation of a cysteine group that is critical for DNA binding [30], [31]. Moreover, we [12] and Johansson and colleagues [32] have shown that *Gclm* −/− mice are not necessarily more sensitive to the pulmonary toxicants diesel exhaust particulate (DEP) or ozone, respectively. This is presumably because of compensatory up-regulation of alternative antioxidant and anti-inflammatory pathways [12], [32]. Interestingly, *Gclm +/−* mice appear to have increased susceptibility to inflammation when exposed to diesel exhaust particles (DEP) [12]. As common single nucleotide polymorphisms (SNPs) in the 5′-promoter regions of both *GCLM* and *GCLC* have been associated with various clinical conditions in human populations [33], including lung diseases [34], it is important to understand the role of GSH synthesis in mediating deleterious effects following exposure to environmental toxicants. Humans who have certain common *GCLM* and *GCLC* genetic polymorphisms have relatively normal GSH levels, but appear to be unable to rapidly synthesize *de novo* GSH in response to oxidative challenge, apparently because of compromised induction of *GCLM* and *GCLC* mRNAs that normally occurs under conditions of oxidative stress. This phenotype is similar to what we observe in *Gclm* +/− mice, in that they demonstrate relatively normal levels of GSH, but seem to be unable to adequately synthesize *de novo* GSH following oxidative challenge. Due to these observations, we believe that our data supports the contention that humans with SNPs within *GCLM* and *GCLC* may demonstrate heightened sensitivity to environmental toxicants that cause oxidative stress.

Although the *Gclm* +/− mouse represents a model that is more clinically relevant to the human condition of *GCLM* SNPs, the *Gclm −/−* mouse provides a unique model to investigate the effect of severely compromised *de novo* GSH synthesis on mounting an inflammatory response following QD challenge. Our results suggest that *Gclm* −/− mice, despite severely reduced GSH levels, do not mount an inflammatory response to intranasal QD treatment whereas this response does occur in *Gclm +/+* and *Gclm +/−* mice.

In order to examine the possibility that the lack of neutrophils in the BALF in *Gclm* −/− mice may be due to interference with cytokine/chemokine signaling, we determined the mRNA levels of a number of such factors using qRT-PCR. These data suggest that the innate immune response in these mice may in fact be compromised. Specifically, the mRNA expression of *GMCSF*, *MCP1* and *IL1β* appears to be muted in *Gclm* −/− mice following TOPO-PMAT QD exposure, relative to *Gclm* +/− and *Gclm* +/+ mice (Figure 5). In addition, the protein levels of KC and TNFα were also unaffected by TOPO-PMAT QD exposure in *Gclm* −/− mice (Figure 6). Diminished expression of these acute-phase early-response proteins may prevent the downstream events necessary for neutrophil recruitment and infiltration. In this regard, we have shown that MMP-dependent shedding of KC bound to the extracellular matrix protein syndecan-1 is a crucial signal for neutrophil migration into the alveolar spaces [35], [36]. Similarly, TNFα is known to be shed by TNFα converting enzyme (TACE; also know as ADAM17) following activation of pro-inflammatory signaling pathways [37], [38]. Consistent with these observations and the presently reported neutrophil influx data, both KC and TNFα were elevated in the *Gclm* +/− and −/− mice. However, MMP activity was not significantly altered with QD exposure, regardless of genotype. Interestingly, the lower levels of neutrophils in the BAL fluid of *Gclm* −/− mice were in agreement with MPO activity in the lung tissue of these mice as it was also diminished relative to both *Gclm* +/+ and +/− mice. Had the levels not been lowered, we may have concluded the neutrophils were recruited to the lungs in Gclm −/− mice but had not migrated to the alveolar space.

An alternate mechanistic explanation for the lack of neutrophil influx into the BALF of *Gclm* −/− mice is that these mice clear the TOPO-PMAT QD more rapidly from their lungs such that a threshold necessary for pro-inflammatory signaling is not achieved. Data from the kinetic modeling analysis would indicate that, indeed, a more rapid clearance of QDs (as indicated by Cd levels in the lung tissue) is evidenced in *Gclm* −/− mice. The mechanism for enhanced clearance is unknown but may be related to more rapid transit of the QDs out of the lung and into either the pulmonary circulation or the draining lymphatic circulation. QDs are known to accumulate in the lymph system [36] and while speculative, it may be that the *Gclm* −/− mice have more motile QD-laden macrophages or more rapid interstitial fluid transport of QD to the thoracic lymph nodes. Regarding direct transport of QD into the circulation, we were unable to find evidence of QD deposition in organs of the reticulo-endothelial system known to sequester QDs such as the liver, spleen, and kidney at any time points examined (data not shown). More rapid mucociliary clearance of the QD from the lungs of *Gclm* −/− mice may also contribute to enhanced clearance out of the lung and bronchial tree. Indeed, GSH is known to play a pivotal role in gap junctional intercellular communication [39], [40], a process important for coordinating ciliary function in respiratory epithelium [41]. Mice lacking GSH may have compensated for the loss of anti-oxidant capacity by up-regulating alternate pathways resulting in more rapid ciliary beat frequency. It is therefore possible that the *Gclm* −/− mice simply clear QDs via mucociliary transport more rapidly than *Gclm +/+* and *Gclm +/−* mice.

In conclusion, mice with normal or slightly diminished capacity for GSH synthesis are susceptible to the pro-inflammatory potential of TOPO-PMAT exposure via nasal instillation. Because there are relatively common functional genetic polymorphisms in the human *GCLM* gene that have been associated with partially compromised GSH synthesis capacity, caution is warranted when considering human exposure scenarios to such nanomaterials. Conversely, the attenuated GSH synthesis capacity in *Gclm −/−* mice is associated with a diminished ability to mount an inflammatory response and/or a more rapid clearance of QDs from the lung. These findings suggest that people with severely compromised GSH status may also fail to respond to such exposures especially if there is up-regulation of compensatory factors similar to those occurring in this mouse model. The mechanisms responsible for this unexpected result are currently under investigation as well as the long term consequences associated with exposure to QDs.

## Supporting Information

Table S1 Primers and probes for fluorescent 5′nuclease quantitative PCR assays are provided online in the Supporting Information file.
(DOCX)
