# Supplementary material for: The Glutathione Synthesis Gene Gclm Modulates Amphiphilic Polymer-Coated CdSe/ZnS Quantum Dot–Induced Lung Inflammation in Mice
Source: PLoS One. 2013 May 27;8(5):e64165. doi: 10.1371/journal.pone.0064165 (PMC3664581; doi:10.1371/journal.pone.0064165)
Supplement: Table S1 — Primers and probes for fluorescent 5′nuclease quantitative PCR assays are provided online in the Supporting Information file. (DOCX) [file pone.0064165.s001.docx]

**Table S1: Primers and probes for fluorescent 5’nuclease quantitative PCR assays.**

| Gene | Primers | Probe |
| --- | --- | --- |
| *Gapdh* | Primer sequences are proprietary to the Applied Biosystems TaqMan Assay-On-Demand gene expression assay. | 6FAM-5'-TGAACGGATTTGGCCGTATTGGGCG-3'-NFQ |
| *Mcp1* | Primer sequences are proprietary to the Applied Biosystems TaqMan Assay-On-Demand gene expression assay. | 6FAM-5'- GGCTCAGCCAGATGCAGTTAACGCC -3'- NFQ |
| *Il1β* | Primer sequences are proprietary to the Applied Biosystems TaqMan Assay-On-Demand gene expression assay. | 6FAM-5'- TGTGCAAGTGTCTGAAGCAGCTATG -3'- NFQ |
| *Gmcsf* | Primer sequences are proprietary to the Applied Biosystems TaqMan Assay-On-Demand gene expression assay. | 6FAM-5'- TTCTCCTTCAAGAAGCTAACATGTG -3'- NFQ |
| *Tnfα* | Primer sequences are proprietary to the Applied Biosystems TaqMan Assay-On-Demand gene expression assay. | 6FAM-5'- CCAGACCCTCACACTCAGATCATCT-3'- NFQ |
| *Gclc* | FP: 5’-ATGTGGACACCCGATGCAGTATT-3’  RP: 5’-TGTCTTGCTTGTAGTCAGGATGGTTT-3’ | 6FAM-5’-CCTAAAGCTAATTAAGAAGAGAGC-TAMRA-3’ |
| *Gclm* | FP: 5’-GCCACCAGATTTGACTGCCTTT-3’  RP: 5’-CAGGGATGCTTTCTTGAAGAGCTT-3’ | 6FAM-5’-TCTGAGGCAAGTTTCCA-TAMRA-3’ |
| *Mt1* | FP: 5’-GCGCCGCGGACAAGT-3’  RP: 5’-TGGGTTGGTCCGATACTATTTACA-3’ | 6FAM-5'-ATGTGACGAACAGCGCTGCCACC-TAMRA-3’ |
| *Mt2* | FP: 5’-TGCAAACAATGCAAATGTACTTCCT-3’  RP: 5’-AGCAGCTGCACTTGTCGGAA-3’ | 6FAM-5'-TGGGAGCACTTCGCACAGCCCA-TAMRA-3’ |
| *Hmox1* | FP: 5’-CCCCAAAACTGGCCTGTAAAA-3’  RP: 5’-GAAATGTCTGGAAACGGATATCAAAG-3’ | 6FAM-5'-CCATGTTGACTGACCACGACTGCTGTCC-TAMRA-3’ |

6FAM: 6-carboxy-fluorescein

TAMRA: 6-carboxy-tetramethyl-rhodamine

NFQ: non-fluorescent quencher
